# Automatic analysis of eyelid movement in de-novo Parkinson’s disease

**DOI:** 10.1038/s41531-025-01021-z

**Published:** 2025-06-06

**Authors:** Louis Kälble, Tereza Tykalova, David Zogala, Petr Dusek, Jan Rusz, Michal Novotny

**Affiliations:** 1 Department of Neurology and Centre of Clinical Neuroscience, First Faculty of Medicine, Charles University, Prague, Czech Republic; 2 Department of Circuit Theory, Faculty of Electrical Engineering, Czech Technical University in Prague, Prague, Czech Republic; 3 Institute of Nuclear Medicine, First Faculty of Medicine, Charles University and General University Hospital in Prague, Prague, Czech Republic

**Keywords:** Biomarkers, Parkinson's disease

## Abstract

This study presents an automated, objective method for eyelid movement assessment in de-novo Parkinson’s disease(PD) using a one-dimensional camera setup during monologue. These measurements were related to Dopamine Transporter Single Photon Emission Tomography and clinical scores. State-of-the-art computer-vision technologies and deep-learning neural networks were utilized to measure fourteen eyelid movement markers describing blinking and eyelid kinematics. Video-recordings were collected from a total of 120 de-novo patients with PD and 55 healthy controls. Abnormal blinking was present in 38% of PD, indicated by a reduced blink rate (*p* < 0.001), an increased inter-blink interval (*p* < 0.001), and an increased rigidity of the palpebral aperture (*p* < 0.001). The classification experiment reached an area under the curve of 0.81 on a blinded test set. The blink rate correlated with the loss of nigral dopaminergic neurons (r = 0.35, *p* < 0.001). These findings suggest eyelid movement markers as strong reflections of striatal dopaminergic activity levels, underscoring the method’s potential as a reliable early PD biomarker.

## Introduction

A decrease in blinking frequency and a loss of facial expression, both characteristic symptoms of Parkinson’s Disease (PD), have been historically associated with bradykinesia, a consequence of reduced dopaminergic activity. Nonetheless, the pathophysiology of eyelid movement is multifaceted, involving multiple cortical and subcortical areas that regulate spontaneous, reflexive, or voluntary movement^1^. Previous research indicates that PD alters spontaneous blink rates and potentially reduces blink amplitude, while blink velocities remain unaffected. Further, the interphase between the closing and opening phase is prolonged, and reflex blinking exhibits hyperexcitability^2–5^. Notably, dopamine therapy or sub-thalamic nucleus deep brain stimulation stabilize blinking in PD, thereby linking the disturbance of basal ganglia circuits closely to the root cause of blinking abnormalities, and thus suggesting eyelid movement as a powerful and easy-to-evaluate indicator for the integrity of nigrostriatal dopaminergic systems^6–8^.

Despite these findings, the current assessment of eyelid movement behavior in PD predominantly relies on subjective evaluation by human raters or complex and costly experimental setups, including electrooculography, electromyography, infrared cameras, or the use of magnetic search coils attached to the eye^3,4,7^. Such setups are hardly applicable in broad clinical practice. Yet, advancements in computer vision technology that gave rise to facial landmark tracking, motivate the development of affordable, objective, video-based methods for facial feature analysis. However, previous video-based methods were designed specifically for facial bradykinesia in PD^9–11^ and do not provide a comprehensive analysis of eyelid movement behavior. Whether the automated video-based analysis of eyelid movement has diagnostic potential in PD remains unexplored. Specifically, given the rise of remote communication via microphone/camera, eyelid movement analysis during common spontaneous speech has the unique potential to be widely applied as a screening instrument to detect early PD or even monitoring treatment efficacy and disease progression. Therefore, our study aims to develop an automated, objective method utilizing state-of-the-art computer-vision technologies and deep-learning neural networks to assess eyelid movement behavior in patients with de-novo, drug-naive PD from a one-dimensional camera setup during freely spoken monologue. Furthermore, we also aim to utilize the objective markers derived from our proposed methodology in conjunction with clinical and dopamine transporter imaging data to enhance the understanding of the eyelid movement disruption in PD and its underlying pathophysiology.

## Results

### Participants

This study included a total of 120 patients with de-novo PD. Of these participants, 39 were female (32.5%) and 81 were male (67.5%). The PD group had an average age of 60.0 years (SD 11.5, range 36–83) and an average duration of motor symptoms of 1.9 years (SD 1.8, range 0.3–11.3). The examination of motor disability according to the MDS-UPDRS III resulted in an average score of 29.0 (SD 11.8, range 6–63). A summary of further clinical information regarding the PD group can be found in Table 1. As a control group, we included 55 sex- and age-matched individuals, consisting of 19 females (34%) and 36 males (66%). Their average age was 60.4 years (SD 9.6, range 37–81). All PD and control participants were Caucasian.

**Table 1:** Demographic and clinical information for patients with PD and healthy controls

	PD (*n* = 120, 39 women)	HC (*n* = 55, 19 women)	*p*-value	*t*-value
*General*				
Age (years)	60.0 (11.5, 36–83)	60.4 (9.6, 37–81)	0.82	−0.23
Sex Ratio	0.68	0.65	–	–
Symptom duration (years)	1.9 (1.8, 0.3–11.3)	–	–	–
*Motor Manifestations*				
MDS-UPDRS III score	29.0 (11.8, 6–63)	3.6 (3.4, 0–13)	<0.001	15.67
MDS-UPDRS 3.2, Facial Item	1.5 (0.81, 0–3)	0.24 (0.43, 0–1)	<0.001	11.25
*Non-Motor Manifestations*				
MoCA	24.9 (3.8, 15–30)	26.3 (1.9, 23–30)	<0.01	−2.80
*Neuroimaging (DAT-SPECT)*				
Mean Putamen SBR	1.47 (0.34, 0.80–2.49)	–	–	–
Mean Posterior Putamen SBR	1.02 (0.30, 0.52–2.17)	–	–	–
Putamen % interhemispheric Difference	0.18 (0.11, 0–0.5)	–	–	–
Posterior Putamen interhemispheric % Difference	0.26 (0.15, 0–0.65)	–	–	–

Values listed in the format mean (standard deviation, range). The neuroimaging values depict the average between hemispheres of the overall and posterior putamen or the percentage difference of values between the two hemispheres.

*PD* Parkinson’s Disease, *MDS-UPDRS III* Movement Disorder Society Unified Parkinson’s Disease rating scale III, *MoCA* Montreal cognitive assessment, *DAT-SPECT* Dopamine transporter single-photon emission computed tomography, *SBR* specific binding ratio.

### Intra-individual variability

Intra-individual variability analysis revealed significant correlations between all markers in the first and second video snippet, except for the relative inter-blink interval deviation marker. The mean absolute intra-individual differences were smaller than the mean absolute inter-individual differences (Supplementary Table 1).

### Between-group differences

**Fig. 1:**
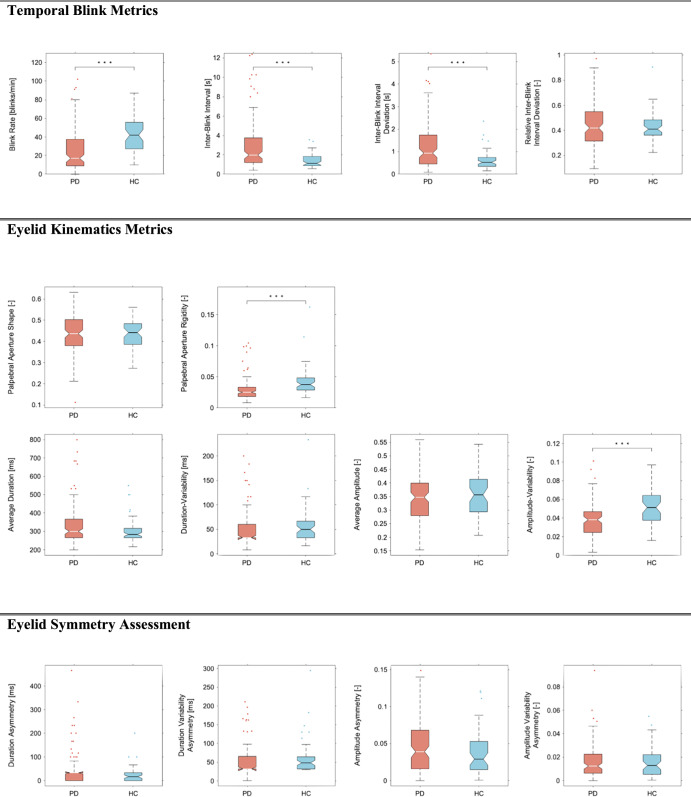
Between-group differences for each blink marker. The centerline in each figure denotes the group median, bounding boxes represent the 25th and 75th percentile. Whiskers denote the non-outlier range of the data and circles denote outliers. To improve the readability, outliers that extend far beyond the non-outlier range are not depicted. Statistically significant group-differences after Bonferroni adjustment are denoted by asterisks: ***p < 0.001, *p < 0.05. PD Parkinson’s disease, HC healthy control.

Our analysis revealed significant statistical differences for multiple blinking characteristics including for the temporal blink metrics markers, the blink rate (*z* = −5.21, *p* < 0.001), the inter-blink interval (*z* = 4.46, *p* < 0.001) and the inter-blink interval deviation (*z* = 4.03, *p* < 0.001). Additionally, we found significant differences in the palpebral aperture rigidity marker (*z* = −4.91, *p* < 0.001) and in the amplitude variability marker (*z* = −4.28, *p* < 0.001). An illustration of between group differences is shown in Fig. 1.

To exclude potential effects of medication on blinking characteristics, we compared patient groups using antihistamine drugs or antidepressants and those not using these medications. In the PD patient group, 17 participants reported using anti-depressant medications and 5 participants reported the use of anti-histaminergic drugs. No significant differences between the medicated and non-medicated patient group with PD were observed except for the palpebral aperture rigidity marker in the antidepressant group not corrected for multiple comparison (*z* = −2.12, *p* < 0.05) (Supplemental Table 2).

### PD diagnostic sensitivity based on abnormal eyelid movement

The best performing combination of blink markers, based on the training data, consisted of five blink markers, and yielded an overall AUC of 0.79 with an accuracy of 72.9% (with a sensitivity of 75.0% and a specificity of 68.2%). The markers included in this grouping were the inter-blink interval (AUC = 0.69), the duration variability marker (AUC = 0.58), the amplitude variability marker (AUC = 0.67), the duration variability asymmetry marker (AUC = 0.52), and the amplitude variability asymmetry marker (AUC = 0.48).

**Fig. 2:**
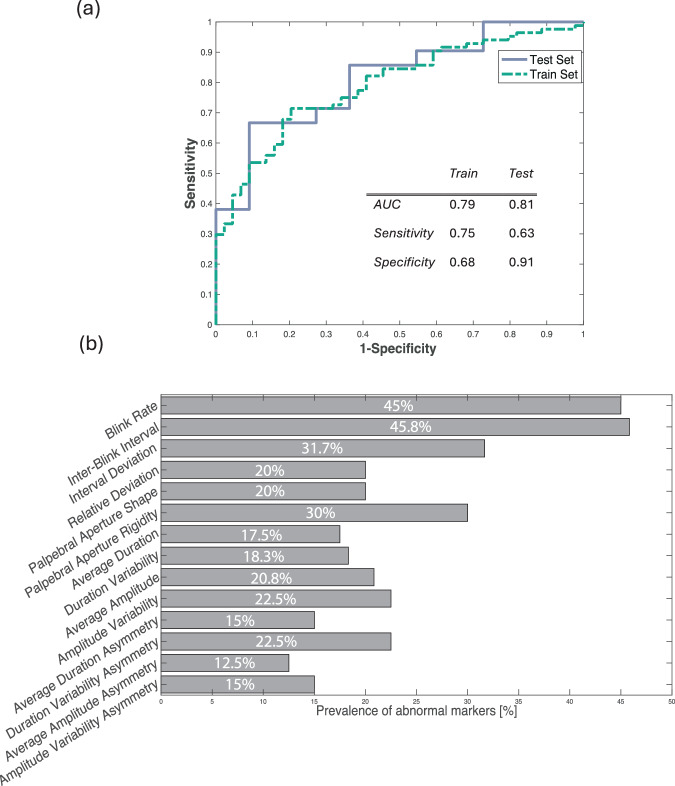
Results of Parkinson’s disease sensitivity analysis based on abnormal eyelid movement. **a** Performance characteristics and the receiver operating characteristic curve of the best performing model for distinguishing between a Parkinson’s disease- and healthy control group. **b** Prevalence of abnormal markers in the Parkinson’s disease group. AUC area under the curve.

Validation of the diagnostic accuracy of the best-performing markers on the blind test set resulted in an AUC of 0.81, with an accuracy of 71.4% (a sensitivity of 62.5% and specificity of 90.9%). The receiver operating characteristic curve of both training and test data sets can be seen in Fig. 2a. The performance of single-marker models can be found in Supplementary Table 3. Based on the receiver operating characteristic curve, the prevalence of abnormal blink behavior in PD, evaluated by an automatic, video-based assessment, is 38.1% while maintaining a false-positive rate under 5%. The automatic assessment correctly identified 15 out of 24 patients with PD of the test set and 10 out of 11 healthy controls. The results of the multicollinearity analysis showed no presence of multicollinearity indicated by no Condition Index value close to the threshold of 10 (Supplementary Table 4).

To further analyze the prevalence of abnormal blink behavior and eyelid movement in PD, we defined blink marker values above the 95^th^, or below the 5^th^ percentile of the control group as abnormal. From these cutoffs, we found that 50 patients with PD (41.6%) have an abnormally low blink rate while 4 patients (3.3%) exhibit and abnormally high blink rate. Upon further investigation, no clear difference in demographic or clinical markers of these patients could be found. Complete demographic and clinical information of the patients with PD that exhibited an abnormally high blink rate are listed in Supplementary Table 5. Upon further investigation we found a total of 67 patients with PD (55.8%) that show one or more abnormal blink markers within the group of temporal blink metrics, 84 (70%) patients with PD that have at least one abnormal marker from the eyelid kinematics metrics group and a total of 58 (48.3%) patients that show abnormal markers from the eyelid asymmetry assessment group. Moreover, the most prevalent abnormal blink characteristics were the blink rate (45%), the inter-blink interval (45.8%) and the palpebral aperture rigidity (30%) (Fig. 2b, Supplementary Table 6).

### Comparison between automated and perceptually evaluated blink rate

Comparing perceptual evaluation and the automated blink rate marker, we detected a significant correlation (*r* = 0.95, *p* < 0.001) between both methods. The diagnostic performance of the model based on perceptual evaluation resulted in an AUC of 0.70, while the automated blink rate marker reached an AUC of 0.74. Sensitivity, Specificity and Accuracy of both models showed the same results (Supplementary Table 7).

### Relation to clinical and imaging parameters

With regards to clinical examination, we detected significant correlations between the total MDS-UPDRS III score and the blink rate (*r* = −0.37, *p* < 0.001), the average inter-blink interval (*r* = 0.25, *p* < 0.01), the average variability of inter-blink interval (*r* = 0.31, *p* < 0.001), and the palpebral aperture rigidity marker (*r* = −0.35, *p* < 0.001). We further found significant correlations between the MDS-UPDRS III facial item 3.2 and the blink rate (*r* = −0.38, *p* < 0.001), the average inter-blink interval (*r* = 0.29, *p* < 0.01), the average variability of inter-blink interval (*r* = 0.29, *p* < 0.01), the palpebral aperture rigidity marker (*r* = −0.40, *p* < 0.001), the average blink amplitude marker (*r* = −0.19, *p* < 0.05), and the average blink duration asymmetry marker (*r* = 0.23, *p* < 0.05). There were no significant correlations between the eyelid movement markers and the MoCA scores.

In the analysis of relationship between the blink markers and the DAT-SPECT markers of the overall and posterior putamen, considering the mean SBRs from both hemispheres, we revealed significant correlations between the putamen and the blink rate (*r* = 0.32, *p* < 0.001), the average inter-blink interval (*r* = −0.30, *p* < 0.01), the variability of inter-blink interval (*r* = −0.23, *p* < 0.05), and the palpebral aperture rigidity (*r* = 0.30, *p* < 0.01). For the posterior putamen, we found significant correlations with the blink rate (*r* = 0.35, *p* < 0.001), the average inter-blink interval (*r* = −0.32, *p* < 0.001), the average variability of the inter-blink interval (*r* = −0.26, *p* < 0.01), the palpebral aperture shape (*r* = −0.21, *p* < 0.05), the palpebral aperture rigidity (*r* = 0.28, *p* < 0.01) and the average blink amplitude (*r* = −0.25, *p* < 0.01).

The correlations between the blink markers and the percent interhemispheric difference between DAT-SPECT SBR of the posterior putamen revealed a weak relationship between the average blinking amplitude asymmetry and the posterior putamen percent difference (*r* = 0.23, *p* < 0.05). A complete listing of all tested correlations can be found in Supplementary Table 8.

## Discussion

This is the first study to explore the potential of a single camera-based video analysis, detecting the disruption of eyelid movement in patients with PD. From video recordings of natural and unconstrained monologues in a large cohort of de-novo patients with PD, we were able to investigate and quantify temporal blink metrics as well as eyelid kinematics and movement symmetries in the blink execution. Our findings unravel the relationship between blinking, clinical and dopamine transporter imaging data, thereby confirming blinking behavior as a strong and accessible indicator of dopaminergic nigrostriatal system integrity. Furthermore, we were able to classify de-novo patients with PD based solely on eyelid movement with an accuracy of 73% on a training set and 71% on a blind test set, indicating a high reliability of our approach for unseen data. The accessibility of this setup enables new opportunities for potential at-home screening prior to a first clinical evaluation. It also provides a possible easy monitoring method for disease progression or treatment effect as both have been shown to be reflected by blinking behavior^6,7,12^.

The best performing combination of eyelid movement markers used in the training of the logistic regression model led to an overall ability to distinguish newly diagnosed patients with PD from a healthy control group with an AUC of 0.81 on a blinded test set. A previous study that utilized perceptual assessment of spontaneous blinking rate during conversation^8^ based on 20 patients with an average disease duration of 6.2 years reported a sensitivity of 65% and a specificity of 83% using a cut-off value of 20 blinks/min for classification^8^. In comparison, our model achieved a similar sensitivity of 63% and higher specificity of 91%, however, at the time of diagnosis and on a blinded test set. These findings are further supported by our comparison of automated and perceptually evaluated blink rate, which evaluated the diagnostic performance of two logistic regression models – each trained using one of the methods. Both models demonstrated the same sensitivity of 74%, specificity of 52% and accuracy of 67%. However, the model based on the automated blink rate marker achieved a slightly higher AUC (0.74 vs 0.70). Together, these results highlight the potential of an automatic approach as a powerful alternative to perceptual evaluation in PD diagnosis.

Interestingly, two markers of the presented model belong to the eyelid kinematics metrics group and two to the eyelid symmetry assessment group with only one stemming from the overall best individually performing group of temporal blink metrics. This may be explained by possible strong multicollinearity between markers such as the inter-blink interval, inter-blink interval deviation and the blink rate, leading to an overall worse performance when combined. We may hypothesize that the remaining markers likely extend the model’s ability to capture different characteristics with no added multicollinearity. These results suggest that every marker group provides important information end extends the description of eyelid movement behavior. Our results further underpin the inclusion of eyelid movement behavior analysis in a multimodal classification model as a powerful upper-face measure, supported by its high specificity but moderate sensitivity for a possible diagnosis^8^.

In accordance with previous literature, our data confirmed an abnormally low spontaneous blink rate in patients with PD, as reflected in the blink rate marker, the inter-blink interval marker and the inter-blink interval variability marker^3,8,13,14^. Furthermore, we also found a small proportion of the PD cohort (3.3%) that exhibited an abnormally elevated spontaneous blink rate. This phenomenon has been previously described as a result of late-disease stages or dopaminergic treatment and was often suggested to be a form of muscle dystonia^3,15^, which is not applicable to our cohort. Indeed, studies that included patients on dopaminergic medication and later disease stages reported a higher ratio of elevated blink rates; Korosec et al. reports one third of patients with an elevated blink rate. Even though the ratio detected by our approach is significantly smaller, the reason behind elevated blink rate in drug naïve de-novo patients with PD is unclear and warrants further investigation. Considering the stability of blink timing, the relative inter-blink interval variability, did not reveal significant differences between PD and HC groups with both exhibiting an average variability of around 40% of the total interval. This may suggest that PD merely prolongs the inter-blink interval, while the relative distance between their occurrence and therefore the overall blink pattern, remains intact. A similar prolongation has been previously reported in voluntary blinking analysis, where the transition between the opening and closing phases was significantly extended in PD, hinting at a potential shared cause^4^. One possible interpretation of this may be to view the reduced blink rate as manifestation of upper-face bradykinesia disrupting only the inter-blink intervals, while the reflexive movement during an actual blink is spared and its speed remains unchanged^5^.

Regarding only the inter-blink intervals, our results suggest that patients with PD exhibit less variability in their palpebral aperture, and a lower variability of blinking amplitudes. Because the amplitude variability is mainly reflected by the width of the palpebral aperture prior to the blink, both markers may be linked to an increased rigidity and bradykinesia of the palpebral aperture in PD. While previous studies reported difficulties in finding a relation between the blink rate and facial bradykinesia^16^, our finding suggests that a reduction of these dynamic blink markers reflects the typical bradykinesia of the upper face in patients with PD and a reduced emotion expressivity manifested as the decrease in spontaneous widening or narrowing of the palpebral aperture^5^. Moreover, while no significant difference in the average shape of the palpebral aperture has been found, the increased bradykinesia may precede widened palpebral apertures and a staring look commonly present in later disease stages^17^.

The analysis of relationships revealed significant correlations between blink markers capturing an abnormally low blink rate and eyelid bradykinesia with the overall motor performance of the patients with PD, suggesting a common pathophysiology with other motor disabilities in PD. This is further supported by our results regarding the relation between DAT-SPECT binding ratios and eyelid movement markers. We revealed significant correlations between the blink rate and related markers and the bradykinesia of the palpebral aperture with both the overall and posterior putamen SBRs. Moreover, we found negative correlations between the posterior putamen SBR and the palpebral aperture shape as well as the average blink amplitude. This suggests an increased palpebral aperture and therefore bigger blink amplitudes with a smaller binding ratio as typically emerging in more severe degeneration of the substantia nigra^17^. In a previous neuroimaging study, blinking abnormalities were similarly reported to correlate with lower gray matter volume in the putamen and caudate nucleus^18^. Moreover, our findings resonate with the notion that the posterior putamen is more severely affected in early PD, showing a higher decrease of the binding ratio and thus a higher sensitivity to disrupted eyelid movement when compared to the overall putamen^19–21^.

Interestingly, we found a positive correlation between the interhemispheric difference of the posterior putamen SBRs and the blink amplitude asymmetry, suggesting that a higher lateral asymmetry in the blink amplitude may be associated with an asymmetrical degeneration of the substantia nigra. Similarly to our findings, homolateral facial bradykinesia (hemihypomimia) has been observed in PD. It is typically more pronounced on the lower part of the face, which may be attributed to the bilateral innervation of the upper part of the face, meaning that the contralateral damage is partially compensated by the less affected ipsilateral hemisphere^22^. Previously, Özekmekçi et al. (2017) found a prevalence of hemihypomimia in up to 6% of 204 patients with PD in Hoehn and Yahr stage II and reported that it tends to occur predominantly in early-onset PD and at a shorter disease duration. However, recent research by Guerra-Hiraldo et al. (2023) revealed a significantly higher prevalence of hemihypomimia in 46% of a cohort of 46 patients with PD (Hoehn and Yahr stages I and II). Using perceptual evaluation, their results suggest that this symptom is more common than previously acknowledged^23^. This is consistent with the significant, albeit low-strength, correlations we found based solely on the upper facial areas in our study. Automated, objective methods to analyze hemihypomimia in PD could further uncover subtle differences, imperceptible to the human eye, and remain an open area of exploration.

The strength of this study lies in its ability to accurately assess disrupted eyelid movement and blink characteristics in PD using a standard one-dimensional camera setup with a 30 FPS temporal resolution. One potential limitation of this study is that our database consisted of only Czech native speakers of white ethnicity, and the analysis was not tested on participants with different ethnic backgrounds. However, the facial landmark tracking solution was trained on a 30k in-the-wild mobile phone camera photos taken from a wide array of different lenses and under various lighting situations^24^, thus the face tracking should yield similar unbiased results for all ethnicities. Moreover, all markers are based on a relative ratio of landmark distances which decreases the effect of distinct ethnic attributes. A second limitation is the inclusion of patients with PD who take medications that may potentially affect blinking behavior, such as anti-depressant or anti-histaminergic drugs. However, this effect is likely minimal, as no patient took medication directly before the examination, meaning only long-term effects of the medication may influence the results. Additionally, there were no significant differences in blinking characteristics between the medicated and non-medicated patient group with PD aside from the palpebral aperture rigidity marker. This marker, however, showed median values closer to the healthy control group than the non-medicated PD group.

In conclusion, our study introduces an automatic, video-based solution for the easy quantification of multiple aspects of disrupted eyelid movement in PD. This opens the door for the inclusion of specific blink characteristics in the multitude of automized models for orofacial biomarkers in PD. We present a measurable eyelid movement marker that can be linked to upper facial bradykinesia, a connection not previously established and confirm that the blink rate and hypomimia related eyelid movement markers are a strong reflection of striatal dopaminergic activity levels. These findings highlight the markers’ sensitivity, suggesting their potential for inclusion in the emerging biomarker battery for PD. They also support the investigation of eyelid movement in other neurological disorders, particularly those affecting basal ganglia dopaminergic pathways such as progressive supranuclear palsy, multiple system atrophy or Huntington’s disease, which have been shown to exhibit altered blink rates^18,25,26^. Importantly, these conditions should be considered as potential differential diagnoses, especially if a reduced blink rate is observed before a clinically established diagnosis. Moreover, our results lay the groundwork for future longitudinal studies, particularly those focusing on dopaminergic treatment, and may provide valuable insights into the progression of PD. By incorporating voluntary and reflex blinking protocols in future examinations, our proposed methodology could provide an even more comprehensive understanding of eyelid movement disruption in PD. Overall, our findings contribute to the growing body of research on computerized assessment of PD manifestations, offering a novel perspective on the role of eyelid movement and blinking behavior in this complex neurological disorder.

## Methods

### Subjects

This study used patient recordings obtained from the longitudinal *Biomarkers in PD* (BIO-PD) project, which aimed to recruit a large, representative sample of de novo patients with PD. Between 2015 and 2024, a cohort of de-novo, drug-naïve patients with PD was recruited for a comprehensive examination at the Department of Neurology of the General University Hospital in Prague, Czech Republic^27^. All diagnoses of PD were clinically established in accordance with the Movement Disorder Society’s clinical diagnostic criteria^28,29^. The clinical assessment of each patient involved: (i) a structured interview acquiring personal- and medical history, current medication intake and history of drug substance abuse; (ii) a semi-quantitative assessment of PD motor symptoms according to the Movement Disorder Society-Unified Parkinson’s Disease Rating Scale, part III (MDS-UPDRS III)^30^; (iii) and a cognitive examination using the Montreal Cognitive Assessment (MoCA)^31^. Each patient’s disease duration was estimated based on the first self-reported onset of motor symptoms. In addition, we recruited sex- and age-matched healthy controls who underwent the same examination protocol. All diagnoses and clinical evaluations were performed by a neurologist experienced in movement disorders (P.D.).

The exclusion criteria for PD were defined as: (i) having a history of therapy with antiparkinsonian medication, (ii) a history of significant neurological disorders except of PD, (iii) a history of other clinical conditions affecting the eye or facial movements such as conjunctivitis, facial paralysis, hemifacial spasm or stroke, significant morphological changes on brain MRI, including basal ganglia lesions and pronounced white matter lesions (Fazekas >2) or (iv) current or past involvement in any speech or hypomimia therapy. The exclusion criteria for controls were defined as: (i) a history of significant neurological disorders, and (ii) presence of mild cognitive impairment.

All participants provided written informed consent prior to their inclusion. The study received approval from the ethics committee of General University Hospital in Prague, Czechia, and has been performed in accordance with the ethical principles laid down by the Declaration of Helsinki.

### Dopamine transporter imaging

All patients with PD underwent further neuroimaging examination dopamine transporter single photon emission computed tomography (DAT-SPECT) to assess the integrity of nigrostriatal dopaminergic functioning. This examination utilized [123I]-2-b-cabomethoxy-3b-(4-iodophenyl)-N-(3-fluoropropyl) nortropane (DaTscan®, GE Healthcare) as the radiopharmaceutical, following the procedure guidelines of the European Association of Nuclear Medicine^32^ and employing the common acquisition and reconstruction parameters described in detail previously^33^. Semi-quantitative analysis of images was performed using the DaTQUANT V2 software. The specific to non-displaceable binding ratios (SBRs) were determined in the bilateral striatum, caudate, putamen, anterior putamen, and posterior putamen using the formula (nucleus uptake – background uptake)/background uptake with bilateral occipital lobes serving as the background reference region. Mean putamen and posterior putamen SBRs from both hemispheres were analyzed in the current study given the known role of these regions in motor control and the presumed bilateral involvement of the nigrostriatal system in blinking regulation.

### Clinical analysis / Facial expressivity examination

A facial expressivity examination was performed using video recordings obtained with a digital camera (Panasonic Handycam HDR-CX410 – Osaka, Japan), originally part of a comprehensive speech examination. All recordings were made within a consistent 2 h window, between 9:00 and 11:00 AM, under standard indoor lighting conditions comprising a mix of artificial ceiling lights and natural light. The camera was positioned approximately one meter in front of the subject’s face, with the natural light source situated behind the camera, providing frontal illumination. The video recordings consisted of freely spoken monologues on a self-chosen, emotionally neutral topic of approximately two minutes, each edited to a duration of one minute. They were recorded at a resolution of 1920 × 1080 pixels with a frame rate of 30 frames per second in RGB format.

### Computerized analysis of eyelid movement

For the computerized analysis of eyelid movement, we utilized a neural network-based facial landmark detection algorithm to extract 478 3D facial landmarks from each video frame^24^. The facial landmark detection algorithm was implemented in Pycharm Professional 2023.1 (JetBrains s.r.o. Prague, CZ) with Python 3.8. Subsequent blink detection and marker calculation were performed in MATLAB® (Mathworks, Natick, MA, USA). An in-depth description of the blink detection and blink marker evaluation can be found in Supplementary Info 1.

**Fig. 3:**
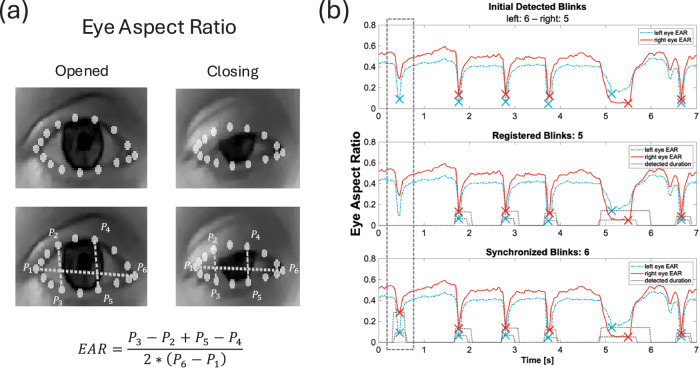
Overview of eye aspect ratio and blink detection algorithm. **a** Computation of the Eye Aspect Ratio in opened and closing eyes. **b** Eye Aspect Ratio over time for both eyes. All three stages/groupings of the blink detection algorithm i.e., the initial detection, registered blinks, and synchronized blinks between both eyes are shown, differences between the three are highlighted. Registered blinks include blinks detected in both eyes with higher confidence.

**Fig. 4:**
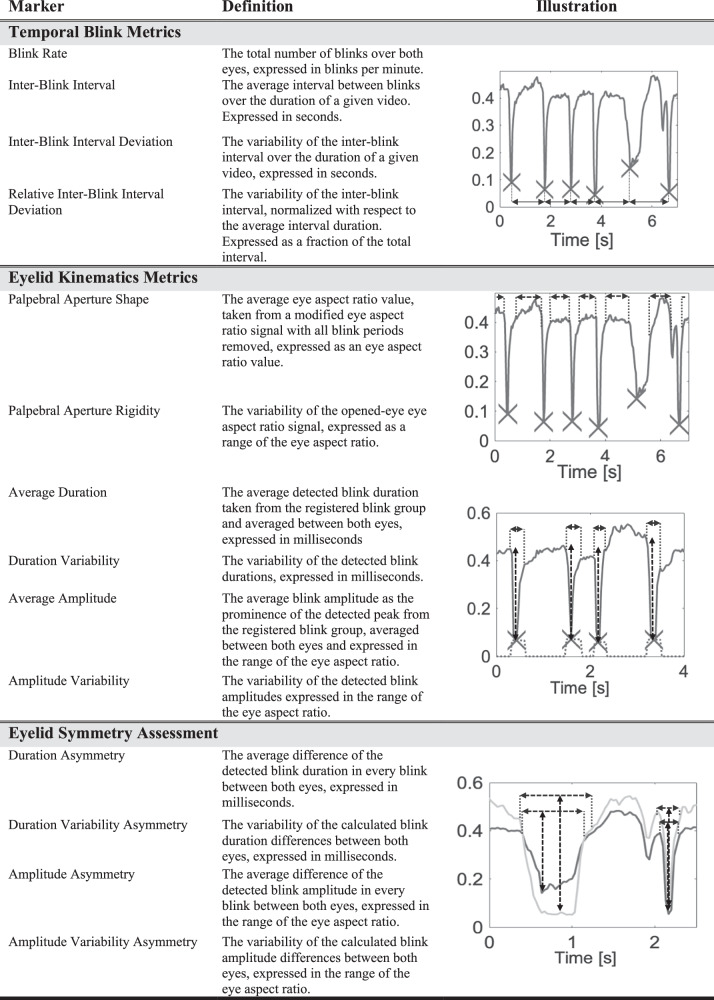
Illustration and description of Eyelid Movement Markers.

We computed a total of fourteen markers capturing various aspects of each patient’s individual eyelid movement characteristics. These markers were based on existing literature and can be categorized into three subdivisions: (i) markers capturing temporal blink metrics such as blink rate and inter-blink intervals; (ii) markers describing eyelid kinematics and (iii) markers assessing left- and right eyelid movement symmetry. The foundation for all markers was the calculation of the eye aspect ratio (EAR), a measure of how shut the eyelids are in relation to the eye’s width, derived from six 3D landmarks in every frame (Fig. 3a). The EAR signal was used for the initial peak detection, which was carried out individually for each eye, capturing blink timing, length, and amplitude. During this step, prolonged eye closures with a duration greater than 2 s were removed. Subsequently, the detected blinks from both eyes were duplicated and underwent registration on one hand and synchronization on the other, creating two different detection vectors more robust against noise. The vector of registered blinks contained exclusively individual well-defined blinks detected simultaneously in both eyes, ensuring a robust analysis of eyelid kinematics, and symmetry of eyelid movement. On the other hand, the vector of synchronized blinks additionally contained blinks that did not have an initial detected counterpart as long as both eyes show a considerable drop in EAR, thus representing a more accurate overall blink rate and general blinking characteristics (Fig. 3b). All marker calculations were performed using non-parametric methods to robustly and accurately capture the characteristics of the data. Therefore, in the following description of the markers, the general term “average” refers to the calculation of the median and the term “deviation” universally refers to the median absolute deviation. A graphical representation of all markers is provided in Fig. 4.

Temporal blink metrics included the *blink rate*, represented as number of blinks per minute, the *inter-blink interval* representing the average value of the duration between two consecutive blinks, the *inter-blink interval deviation* representing deviation in inter-blink intervals, and the *relative inter-blink interval deviation* representing the deviation relative to the average inter-blink interval length.

The eyelid kinematics metrics subgroup included a total of six markers. Two markers captured the shape and rigidity of the palpebral aperture. The *palpebral aperture shape*, calculated as the average EAR value while excluding all blinking phases, the *palpebral aperture rigidity*, calculated as the deviation of this partial signal. The remaining four markers assess kinematic aspects of blinking and included the *average duration* calculated as the average duration of a blink, the *duration variability*, calculated as the deviation of blink durations, the *average amplitude*, calculated as the average blinking amplitude of the detected blinks, and the *amplitude variability*, calculated as the deviation of blink amplitudes.

Lastly, we assessed asymmetries of the blinking process using four markers. *The duration asymmetry*, calculated as the average difference in blinking duration between eyes, the *amplitude asymmetry*, calculated as the average difference of blink amplitudes between both eyes, the *duration variability asymmetry*, calculated from the difference of deviations of blinking duration between both eyes, and the *amplitude variability asymmetry*, calculated as the difference of deviations of blinking amplitudes.

### Statistical analysis

To evaluate statistical differences between groups, we employed the Wilcoxon ranksum test for non-normally distributed data and the student t-test for normally distributed data. Normality was tested using the one-sample Kolmogorov-Smirnov test. To account for multiple comparisons, we set the corrected level of significance to at *α* = 0.0036, corresponding to the Bonferroni adjustment for fourteen markers (0.05/14).

In addition, we analyzed the intra-individual variability of the full dataset by splitting each recording in two 30-s snippets. Pairwise Spearman’s correlation was calculated for each marker across the full dataset and the mean absolute intra-individual and inter-individual differences were computed for each marker.

Correlation analyses between markers and clinical data or neuroimaging data were performed using Spearman’s partial correlation, controlled for age.

Lastly, we analyzed the ability of the markers to distinguish between a healthy population and patients with PD. We trained a multinomial binary logistic regression model by randomly partitioning the data into a stratified 80/20 training and test dataset i.e., with the training set consisting of 96 patients with PD and 44 controls, while the test set contained 24 patients with PD and 11 controls. The training and test splits were balanced with respect to age (*p* = 0.39), sex (*p* = 0.96 for female patients, *p* = 0.93 for male patients), MDS-UPDRS III score (*p* = 0.75 for patients with PD, *p* = 0.92 for controls) and MDS-UPDRS facial item 3.2 score (*p* = 0.09 for patients with PD, *p* = 0.76 for controls). The highest performing combinations of markers were investigated from 10-fold cross-validation on the partitioned training data using grid search. The area under the curve (AUC), obtained from the receiver operating characteristic curve, was used as a measure of diagnostic accuracy. Furthermore, we calculated the accuracy, sensitivity, and specificity as measures of each model’s performance. Markers included in the regression model, based on the results of the 10-fold-cross-validation, were tested for multicollinearity by calculating the eigenvalues from the standardized correlation matrix and computing the Condition Index and Variance Decomposition Proportion.

Finally, we compared the diagnostic performance of the automated blink rate marker with that of a human rater, who performed a perceptual assessment of the dataset. The perceptual assessment involved a manual count of blinks for each recording, played at half speed. Spearman’s rank correlation coefficient was computed to assess the relationship between the two methods. Additionally, the diagnostic performance of two logistic regression models, each trained using 10-fold cross validation on one of the datasets, was evaluated.

## Supplementary information

Supplementary material

STROBE

## Data Availability

Individual participant data that underlie the findings of this study are available upon reasonable request from the corresponding author. The data are not publicly available due to their containing of information that could compromise the privacy of study participants.

## Code availability

The facial landmark detection was performed using a publicly available Python implementation of Google’s Mediapipe FaceMesh library^34^ in the newest version 0.9.1.0.

## References

[1] Bologna, M., Paparella, G., Valls-Solé, J., Hallett, M. & Berardelli, A. Neural control of blinking. *Clin. Neurophysiol.* **161**, 59–68 (2024). 38447495 10.1016/j.clinph.2024.02.023

[2] Kaneko, K. & Sakamoto, K. Spontaneous blinks of Parkinson’s disease patients evaluated by EMG and EOG. *Electromyogr. Clin. Neurophysiol.* **41**, 87–96 (2001). 11284060

[3] Korošec, M., Zidar, I., Reits, D., Evinger, C. & VanderWerf, F. Eyelid movements during blinking in patients with Parkinson’s disease. *Mov. Disord.* **21**, 1248–1251 (2006). 16685691 10.1002/mds.20930

[4] Agostino, R. et al. Voluntary, spontaneous, and reflex blinking in Parkinson’s disease. *Mov. Disord.* **23**, 669–675 (2008). 18175339 10.1002/mds.21887

[5] Bologna, M. et al. Facial bradykinesia. *J. Neurol. Neurosurg. Psychiatry* **84**, 681–685 (2013). 23236012 10.1136/jnnp-2012-303993

[6] Karson, C. N. Spontaneos Eye-Blink Rates and Dopaminergic Systems. *Brain* **106**, 643–653 (1983). 6640274 10.1093/brain/106.3.643

[7] Bologna, M., Fasano, A., Modugno, N., Fabbrini, G. & Berardelli, A. Effects of subthalamic nucleus deep brain stimulation and l-dopa on blinking in Parkinson’s disease. *Exp. Neurol.* **235**, 265–272 (2012). 22366535 10.1016/j.expneurol.2012.02.004

[8] Fitzpatrick, E., Hohl, N., Silburn, P., O’Gorman, C. & Broadley, S. A. Case–control study of blink rate in Parkinson’s disease under different conditions. *J. Neurol.* **259**, 739–744 (2012). 21984191 10.1007/s00415-011-6261-0

[9] Rajnoha, M. et al. Towards Identification of Hypomimia in Parkinson’s Disease Based on Face Recognition Methods. *2018 10th International Congress on Ultra Modern Telecommunications and Control Systems and Workshops (ICUMT)* 1–4, 10.1109/ICUMT.2018.8631249 (IEEE, Moscow, Russia, 2018).

[10] Abrami, A. et al. Automated Computer Vision Assessment of Hypomimia in Parkinson Disease: Proof-of-Principle Pilot Study. *J. Med. Internet Res.* **23**, e21037 (2021). 33616535 10.2196/21037 PMC7939934

[11] Novotny, M. et al. Automated video-based assessment of facial bradykinesia in de-novo Parkinson’s disease. *npj Digit. Med.* **5**, 1–8 (2022). 35851859 10.1038/s41746-022-00642-5 PMC9293947

[12] Peshori, K. R., Schicatano, E. J., Gopalaswamy, R., Sahay, E. & Evinger, C. Aging of the trigeminal blink system. *Exp. Brain Res.* **136**, 351–363 (2001). 11243477 10.1007/s002210000585

[13] Karson, C. N., Lewitt, P. A., Calne, D. B. & Wyatt, R. J. Blink rates in parkinsonism. *Ann. Neurol.* **12**, 580–583 (1982). 7159063 10.1002/ana.410120614

[14] Deuschl, G. & Goddemeier, C. Spontaneous and reflex activity of facial muscles in dystonia, Parkinson’s disease, and in normal subjects. *J. Neurol. Neurosurg. Psychiatry* **64**, 320–324 (1998). 9527141 10.1136/jnnp.64.3.320 PMC2169979

[15] Kimber, T. E. & Thompson, P. D. Increased blink rate in advanced Parkinson’s disease: A form of ‘off’-period dystonia?. *Mov. Disord.* **15**, 982–985 (2000). 11009209 10.1002/1531-8257(200009)15:5<982::aid-mds1033>3.0.co;2-p

[16] Maycas-Cepeda, T. et al. Hypomimia in Parkinson’s Disease: What Is It Telling Us?. *Front. Neurol.* **11**, 603582 (2021). 33569034 10.3389/fneur.2020.603582 PMC7868377

[17] Jankovic, J. Parkinson’s disease: clinical features and diagnosis. *J. Neurol. Neurosurg. Psychiatry* **79**, 368–376 (2008). 18344392 10.1136/jnnp.2007.131045

[18] Bologna, M. et al. Neuroimaging correlates of blinking abnormalities in patients with progressive supranuclear palsy. *Mov. Disord.* **31**, 138–143 (2016). 26636556 10.1002/mds.26470

[19] Pirker, W. et al. Progression of dopaminergic degeneration in Parkinson’s disease and atypical parkinsonism: A longitudinal β-CIT SPECT study. *Mov. Disord.* **17**, 45–53 (2002). 11835438 10.1002/mds.1265

[20] Kägi, G., Bhatia, K. P. & Tolosa, E. The role of DAT-SPECT in movement disorders. *J. Neurol. Neurosurg. Psychiatry* **81**, 5–12 (2010). 20019219 10.1136/jnnp.2008.157370

[21] Matesan, M. et al. I-123 DaTscan SPECT Brain Imaging in Parkinsonian Syndromes: Utility of the Putamen-to-Caudate Ratio. *J. Neuroimaging* **28**, 629–634 (2018). 29905019 10.1111/jon.12530

[22] Özekmekçi, S., Benbir, G., Özdogan, F. Y., Ertan, S. & Kiziltan, M. E. Hemihypomimia, a rare persistent sign in Parkinson’s disease. *J. Neurol.* **254**, 347–350 (2007). 17268884 10.1007/s00415-006-0372-z

[23] Guerra-Hiraldo, J. D. et al. Hemihypomimia in Parkinson’s disease: an under-recognized clinical sign?. *J. Neurol.* **270**, 548–551 (2023). 35925399 10.1007/s00415-022-11292-8

[24] Kartynnik, Y., Ablavatski, A., Grishchenko, I. & Grundmann, M. Real-time Facial Surface Geometry from Monocular Video on Mobile GPUs. arXiv preprint, 10.48550/arXiv.1907.06724 (2019).

[25] Bologna, M. et al. Blinking in patients with clinically probable multiple system atrophy. *Mov. Disord.* **29**, 415–420 (2014). 24532058 10.1002/mds.25830

[26] Fekete, R. & Jankovic, J. Upper facial chorea in Huntington disease. *J. Clin. Mov. Disord.* **1**, 7 (2014). 26788333 10.1186/2054-7072-1-7 PMC4711000

[27] Dušek, P. et al. Clinical characteristics of newly diagnosed Parkinson’s disease patients included in the longitudinal BIO-PD study. *Cesk. Slov. Neurol. Neurochir.* **83**, 633–639 (2020).

[28] Postuma, R. B. et al. MDS clinical diagnostic criteria for Parkinson’s disease: MDS-PD Clinical Diagnostic Criteria. *Mov. Disord.* **30**, 1591–1601 (2015). 26474316 10.1002/mds.26424

[29] Berg, D. et al. Movement disorder society criteria for clinically established early Parkinson’s disease. *Mov. Disord.* **33**, 1643–1646 (2018). 30145841 10.1002/mds.27431

[30] Goetz, C. G. et al. Movement Disorder Society-sponsored revision of the Unified Parkinson’s Disease Rating Scale (MDS-UPDRS): Scale presentation and clinimetric testing results. *Mov. Disord.* **23**, 2129–2170 (2008). 19025984 10.1002/mds.22340

[31] Kopecek, M. et al. Montreal cognitive assessment (MoCA): Normative data for old and very old Czech adults. *Appl. Neuropsychology: Adult* **24**, 23–29 (2017). 10.1080/23279095.2015.1065261 27144665

[32] Darcourt, J. et al. EANM procedure guidelines for brain neurotransmission SPECT using 123 I-labelled dopamine transporter ligands, version 2. *Eur. J. Nucl. Med. Mol. Imaging* **37**, 443–450 (2010). 19838702 10.1007/s00259-009-1267-x

[33] Dušek, P. et al. Relations of non-motor symptoms and dopamine transporter binding in REM sleep behavior disorder. *Sci. Rep.* **9**, 15463 (2019). 31664065 10.1038/s41598-019-51710-y PMC6820530

[34] Google. Face landmark detection guide for Python | Google AI Edge https://ai.google.dev/edge/mediapipe/solutions/vision/face_landmarker/python. (2024)

