# Supplementary material for: Automatic analysis of eyelid movement in de-novo Parkinson’s disease
Source: NPJ Parkinsons Dis. 2025 Jun 6;11:153. doi: 10.1038/s41531-025-01021-z (PMC12144097; doi:10.1038/s41531-025-01021-z)
Supplement: Supplementary file 1 — Supplementary material [file 41531_2025_1021_MOESM1_ESM.docx]

**SUPPLEMENTARY MATERIAL**

**Supplementary Info 1: A detailed description of the video processing pipeline and analytic algorithm.**

**Video Processing**

As a first step, each video was split into separate frames and then individually underwent the facial landmark detection process. All detected landmarks were subsequently used in further preprocessing and the estimation of eyelid movement behavior markers.

**Facial Landmark Detection**

To detect the facial landmarks, the end-to-end neural network based facial landmark detection algorithm “Face Mesh” described in Kartynnik et al (2019) was applied. It provided 3D facial landmark estimations forming a mesh of 468 points from single RGB video frames as its input (see **Supplementary Figure 1**). A machine learning pipeline of two real-time, deep learning neural networks are applied in the process. First, a face detection neural network provides a face bounding box as well as a number of basic landmarks e.g., the center of the eyes or the nose tip. The original frame is then cropped and fed into the second 3D-mesh-prediction neural network predicting the approximate 3D surface via regression and outputting a vector of the desired 3D landmark coordinates^1^. Along with the 3D landmark estimations, the mesh prediction network returns the probabilities of a face being contained within the cropped frame and reasonably aligned. The Face Mesh algorithm has been pre-trained on 30K in-the-wild mobile camera photos taken from a wide range of sensors and under different lighting conditions. Considering the standardized recording conditions during the clinical assessment the 30K in-the-wild provide sufficient sensitivity and robustness for the subsequent feature extraction ^1^.

In addition to the 3D face landmark detection models, another so-called Attention Mesh model is available that further refines landmarks of higher interest areas such as the lips and eyes and adds further ten landmarks describing both irises, bringing the total number of estimated landmarks to 478. At a higher cost of computing, a more precise measurement around these areas can be achieved.

The landmark detection algorithm was executed in the PyCharm Professional environment with Python 3.8 (JetBrains s.r.o.). The described Face Mesh solution provides the option to define a maximum number of faces to track in an image, *max_num_faces*, which has been set to one.

The *refine_landmarks* option has been activated, leading to 478 more robust landmarks that are refined around the mouth- and eye-areas. The script was modified to create a mat-file for each

frame containing all 478 landmarks as 3D coordinates, normalized to the image size, that would subsequently be used for the analysis of eyelid movement and blinking behavior.

| 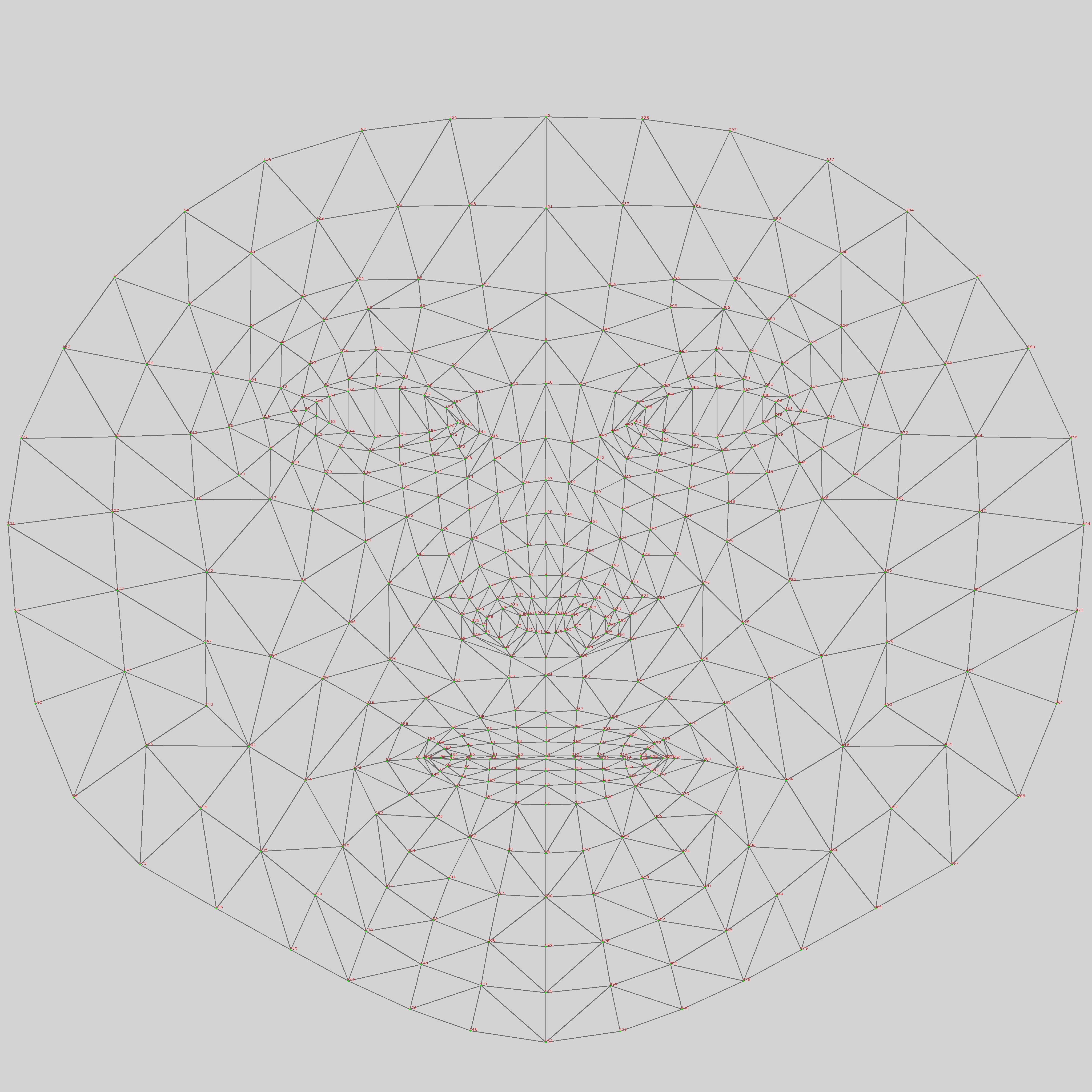 |
| --- |
| Supplementary Figure 1: 468 Facial Landmarks of Google’s Mediapipe Face Mesh Algorithm. |

**Image preprocessing**

If the facial landmark detection failed i.e., the set requirements for tracking- or face presence confidence were not met, or if the head position moved out of frame, no facial landmarks were available for the current frame. However, Face Mesh provides very robust tracking and only a small fraction of frames from all videos showed head movements that exceeded the video frame. Because of that, the exclusion events did not occur often enough to impact the performance and temporal resolution of the subsequent analysis. All missed frames were therefore included in any time-sensitive calculation as an interval of $\frac{1}{30}s=33.3 ms$, while no landmark calculations were performed for this frame.

**Eyelid behavior marker conception and estimation**

All conceptualized markers were created based on dynamic and numeric characteristics of the eye aspect ratio (EAR), measured for each eye in each frame of a video. The EAR is calculated based on 6 facial landmarks and describes the extent of eye openness using the ratio of palpebral aperture to the width of the eye from the following formula:

$$EAR=\frac{P_{vert1,1}-P_{vert1,2}+P_{vert2,1}-P_{vert2,2}}{2*\left( P_{hor2}-P_{hor1} \right)}$$

The Face Mesh landmarks used for the EAR calculation on the left and right side are displayed in **Supplementary Figure 2**.

The marker calculation underlies a detection of blinks based on the curve of the eye aspect ratio. As a first step in the detection, a peak detection is executed individually for both eyes. Here, all detected peaks that have a peak prominence higher than the 80^th^ percentile of detected peak prominences are used in the subsequent processing where double detected blinks (i.e. multiple detected peaks during one long blink) and blinks that last longer than 2 seconds are removed. Next, the two individually detected blink arrays of both eyes are compared and registered onto another using decision logic. Any two blinks, that show a corresponding blink on the other side based on the amplitude, duration and location are saved together in the array of registered blinks. Additional blinks, that do not have an initial detected counterpart, are not saved in the array of registered blinks but in the array of synchronized blinks. For this array, a secondary blink detection is run on the opposite side. This blink detection is run using an appropriate interval around the lone blink and the results are evaluated based on blink amplitude, duration, and location in comparison to the other side, and its own blinks, however less strict than in the first detection to capture even blinks that may be distorted or otherwise noisy. In case a new blink is found, both are kept in the synchronized blinks. In case no new blink is found, the lone blink is deleted from the synchronized blinks indicating a false detection. This concludes the blink detection pipeline which providing an array of precisely detected registered blinks and an all-encompassing array of synchronized blinks. These blinks are then used in the consecutive calculation of fourteen blink markers.

The markers are grouped based on the behavior they capture. The first grouping consists of six markers and describes temporal blink metrics. It includes the following markers:

1. blink rate
2. inter-blink interval
3. interval deviation
4. relative deviation

The *blink rate* describes the total number of blinks in one minute and is calculated from the synchronized blinks to ensure the correct value over both eyes.

The *inter-blink interval* describes the median interval between two blinks from all detected synchronized blinks in seconds.

The *interval deviation* describes the median absolute deviation of all detected synchronized intervals in seconds.

The *relative deviation* describes the variability of inter-blink intervals relative to the average interval length and is expressed as a fraction of the average inter-blink interval.

The next group of markers describe eyelid kinematics metrics of individual blinks. It includes the following four markers:

1. palpebral aperture shape
2. palpebral aperture rigidity
3. average duration
4. duration variability
5. average amplitude
6. amplitude variability

Here, the *palpebral aperture shape* marker is calculated as the median of the EAR values that lie outside of a blink duration. It describes the average openness of the palpebral aperture expressed as the EAR value.

The *palpebral aperture rigidity* marker describes the dynamic changes of the palpebral aperture when the blinking process is left out and is expressed as a range of EAR values

The *average duration* is calculated from the interval of the start and end point of a detected blink peak using a triangular detection method. It describes the total blink duration and encompasses opening and closing phase of the eyelids. It is expressed in milliseconds.

The *duration variability* describes the variability of all detected blink durations and is expressed in milliseconds.

The *average amplitude* is measured as the peak prominence of each detected blink. The marker describes the average amplitude of all blinks and is expressed as the range of EAR values.

The *amplitude variability* describes the variability of all detected blink amplitudes, it is expressed as the range of EAR values.

The last group consists of markers capturing asymmetries of individual blink behaviors. These last four markers are included:

1. average duration asymmetry
2. duration variability asymmetry
3. average amplitude asymmetry
4. amplitude variability asymmetry

The *average* *duration asymmetry* describes the average difference between the left and right eye of all detected blink durations and is expressed in milliseconds.

The *duration variability asymmetry* captures the difference of dynamic behaviors between the left and right eye as the difference of deviations of the two blink durations.

The *average* *amplitude asymmetry* captures the average difference between left and right eye blink amplitudes and is expressed in EAR values

Lastly, the *amplitude variability asymmetry* captures the differences of dynamic behavior of the left and right eye blink amplitudes as the difference of their individual blink amplitude deviations, expressed in EAR values.

| 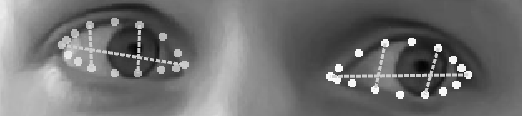 **133**  **33**  **160**  **158**  **144**  **153**  **263**  **362**  **385**  **387**  **380**  **373** |
| --- |
| Supplementary Figure 2: Facial Landmarks required for the calculation of the eye aspect ratio of each side. Numbers indicate the landmark indices of the Face Mesh detection algorithm. |

**Supplementary table 1: Intra Individual Variability Analysis. Correlation analysis results between two 30s videos per participant and mean absolute difference for each marker.**

| **Marker** | ***r*** | ***p-value (corrected)*** | ***Mean Absolute Intra-Individual Difference*** | ***Mean Absolute Inter-Individual Difference*** |
| --- | --- | --- | --- | --- |
| *Temporal Blink Metrics* | | | | |
| Blink Rate [blinks/min] | **0.92** | **<0.001** | 6.66 | 25.69 |
| Inter-Blink Interval [s] | **0.66** | **<0.001** | 1.33 | 3.45 |
| Interval Variability Deviation [s] | **0.47** | **<0.001** | 0.57 | 1.15 |
| Relative Deviation [%] | 0.22 | 0.16 | 0.17 | 0.18 |
| *Eyelid Kinematics Metrics* | | | | |
| Palpebral Aperture Shape [-] | **0.84** | **<0.001** | 0.01 | 0.02 |
| Palpebral Aperture Rigidity [-] | **0.64** | **<0.001** | 46.04 | 93.56 |
| Average Duration [ms] | **0.74** | **<0.001** | 24.35 | 36.16 |
| Duration Variability[ms] | **0.48** | **<0.001** | 0.03 | 0.09 |
| Average Amplitude [-] | **0.88** | **<0.001** | 0.02 | 0.02 |
| Amplitude Variability [-] | **0.49** | **<0.001** | 37.76 | 53.09 |
| *Eyelid Symmetry Assessment* | | | | |
| Average Duration Asymmetry [ms] | **0.48** | **<0.001** | 0.03 | 0.04 |
| Duration Asymmetry Variability [ms] | **0.44** | **<0.001** | 0.01 | 0.02 |
| Average Amplitude Asymmetry [-] | **0.60** | **<0.001** | 6.66 | 25.69 |
| Amplitude Asymmetry Variability [-] | **0.38** | **<0.001** | 1.33 | 3.45 |

**Supplementary table 2: Group-Differences (not corrected) between medicated and non-medicated patient groups with PD**

| **Marker** | ***Anti-Depressant*** | | ***Anti-Histaminergic*** | | ***Non-Medicated*** | | ***Anti-Depressant*** | ***Anti-Histaminergic*** | |
| --- | --- | --- | --- | --- | --- | --- | --- | --- | --- |
|  | *z* | *p* | *z* | *p* | *patient group median* | | | | |
| *Temporal Blink Metrics* | | | | | | | | | |
| Blink Rate [blinks/min] | -0.80 | 0.43 | -1.48 | 0.14 | 17 | 25 | | | 26 |
| Inter-Blink Interval [s] | 0.01 | 0.99 | 1.40 | 0.16 | 2.27 | 1.85 | | | 1.60 |
| Interval Variability Deviation [s] | 0.54 | 0.59 | 1.11 | 0.27 | 0.958 | 0.679 | | | 0.77 |
| Relative Deviation [%] | 0 | 1 | 0.12 | 0.90 | 0.421 | 0.404 | | | 0.418 |
| *Eyelid Kinematics Metrics* | | | | | | | | | |
| Palpebral Aperture Shape [-] | -0.69 | 0.49 | -1.27 | 0.20 | 0.431 | 0.442 | | | 0.484 |
| Palpebral Aperture Rigidity [-] | -2.12 | 0.03 | 0.53 | 0.59 | 0.024 | 0.033 | | | 0.021 |
| Average Duration [ms] | -1.76 | 0.08 | -0.11 | 0.91 | 291 | 300 | | | 283 |
| Duration Variability[ms] | -1.37 | 0.17 | 1.20 | 0.23 | 33.3 | 50 | | | 33.3 |
| Average Amplitude [-] | -1.10 | 0.27 | -0.20 | 0.85 | 0.343 | 0.372 | | | 0.315 |
| Amplitude Variability [-] | 0.29 | 0.77 | -0.31 | 0.76 | 0.037 | 0.038 | | | 0.042 |
| *Eyelid Symmetry Assessment* | | | | | | | | | |
| Average Duration Asymmetry [ms] | -1.11 | 0.27 | -0.26 | 0.79 | 16.7 | 33.3 | | | 33.3 |
| Duration Asymmetry Variability [ms] | 0.10 | 0.92 | 0.75 | 0.46 | 32.3 | 32.3 | | | 32.3 |
| Average Amplitude Asymmetry [-] | 0.54 | 0.59 | -1.38 | 0.17 | 0.039 | 0.032 | | | 0.073 |
| Amplitude Asymmetry Variability [-] | 1.83 | 0.07 | 0.41 | 0.68 | 0.013 | 0.009 | | | 0.012 |
|  |  |  |  |  |  |  | | |  |

**Supplementary table 3: Diagnostic accuracy of single blink markers on the blind test set**

| **Blink Marker** | **AUC** | **Accuracy** | **Sensitivity** | **Specificity** | |
| --- | --- | --- | --- | --- | --- |
| *Temporal Blink Metrics* | | | | |  |
| Blink Rate | 0.76 | 0.69 | 0.79 | 0.45 | |
| Inter-Blink Interval | 0.76 | 0.57 | 0.46 | 0.82 | |
| Interval Deviation | 0.73 | 0.66 | 0.58 | 0.82 | |
| Relative Deviation | 0.52 | 0.49 | 0.54 | 0.36 | |
| *Eyelid Kinematics Metrics* | | | | |  |
| Palpebral Aperture Shape | 0.46 | 0.57 | 0.75 | 0.18 | |
| Palpebral Aperture Rigidity | 0.80 | 0.71 | 0.71 | 0.73 | |
| Average Duration | 0.63 | 0.63 | 0.79 | 0.23 | |
| Duration Variability | 0.57 | 0.51 | 0.50 | 0.55 | |
| Average Amplitude | 0.59 | 0.54 | 0.67 | 0.23 | |
| Amplitude Variability | 0.77 | 0.69 | 0.67 | 0.73 | |
| *Eyelid Symmetry Assessment* | | | | |  |
| Average Duration Asymmetry | 0.61 | 0.57 | 0.63 | 0.45 | |
| Duration Asymmetry Variability | 0.65 | 0.69 | 0.67 | 0.73 | |
| Average Amplitude Asymmetry | 0.48 | 0.46 | 0.58 | 0.18 | |
| Amplitude Asymmetry Variability | 0.52 | 0.49 | 0.38 | 0.73 | |

**Supplementary table 4: Multicollinearity Diagnostics for the multinomial logistic regression model**

| **Eigenvalue** | **Condition Index** | **Variance Decomposition Proportion**  **Multinomial Logistic Regression Model** | | | | |
| --- | --- | --- | --- | --- | --- | --- |
|  |  | *Inter-Blink Interval* | *Duration Variability* | *Amplitude Variability* | *Duration Asymmetry Variability* | *Amplitude Asymmetry Variability* |
| 0.294 | 2.441 | 0.01 | 0.08 | 0.85 | <0.01 | 0.07 |
| 0.512 | 1.849 | 0.46 | 0.05 | 0.03 | 0.01 | 0.46 |
| 0.996 | 1.326 | 0.04 | 0.42 | 0.03 | 0.50 | 0.02 |
| 1.449 | 1.099 | 0.47 | 0.03 | 0.05 | <0.01 | 0.46 |
| 1.750 | 1.000 | 0.02 | 0.43 | 0.05 | 0.50 | <0.01 |

**Supplementary table 5: Demographic and Clinical Information for Patients with PD that exhibit an abnormally high Blink Rate**

| **Patient-Group** | **Sex** | **Age** | **Symptom Duration (years)** | **MoCA** | **MDS-UPDRS III** | **MDS-UPDRS Facial Item 3.2** |
| --- | --- | --- | --- | --- | --- | --- |
| **PD** | M | 62 | 1 | 27 | 20 | 1 |
| **PD** | M | 43 | 5.7 | 26 | 14 | 0 |
| **PD** | F | 66 | 1.8 | 24 | 7 | 0 |
| **PD** | M | 48 | 2.3 | 17 | 19 | 0 |
| **Mean (Sex-Ratio for “Sex”)** | 0.75 | 54.8 | 2.7 | 23.5 | 15 | 0.25 |
| **Standard Deviation** | - | 11.0 | 2.1 | 4.5 | 6.0 | 0.5 |
| **68% Confidence Interval around the PD group mean (Sex-Ratio for “Sex”)** | 0.68 | 48.5-71.5 | 0.1-3.7 | 21.1-28.7 | 18.2-40.8 | 0.69-2.31 |

**Supplementary table 6: Abnormal marker counts of PD participants above and below the normal range**

| **Marker** | **# Below** | **# Above** | **# Total** | **% of total** | |
| --- | --- | --- | --- | --- | --- |
| *Temporal Blink Metrics* | | | | |  |
| Blink Rate | 50 | 4 | 54 | 45 | |
| Inter-Blink Interval | 7 | 48 | 55 | 45.8 | |
| Interval Deviation | 6 | 32 | 38 | 31.7 | |
| Relative Deviation | 9 | 15 | 24 | 20 | |
| *Eyelid Kinematics Metrics* | | | | |  |
| Palpebral Aperture Shape | 6 | 18 | 24 | 20 | |
| Palpebral Aperture Rigidity | 30 | 6 | 36 | 30 | |
| Average Duration | 11 | 10 | 21 | 17.5 | |
| Duration Variability | 16 | 6 | 22 | 18.3 | |
| Average Amplitude | 13 | 12 | 25 | 20.8 | |
| Amplitude Variability | 24 | 3 | 27 | 22.5 | |
| *Eyelid Symmetry Assessment* | | | | |  |
| Duration Asymmetry | 0 | 18 | 18 | 15 | |
| Duration Asymmetry Variability | 20 | 7 | 27 | 22.5 | |
| Amplitude Asymmetry | 10 | 5 | 15 | 12.5 | |
| Amplitude Asymmetry Variability | 11 | 7 | 18 | 15 | |

**Supplementary table 7: Comparison of computerized assessment and perceptual evaluation of the blink rate**

| **Model** | **Spearman’s Correlation Coefficient** | **PD Diagnostic Sensitivity Based on Participants’ Blink Rate** | | | |
| --- | --- | --- | --- | --- | --- |
|  |  | *Sensitivity* | *Specificity* | *Accuracy* | *AUC* |
| *Computerized assessment* | **r = 0.95** | 0.74 | 0.53 | 0.67 | 0.74 |
| *Perceptual evaluation* | **p = <0.001** | 0.74 | 0.53 | 0.67 | 0.70 |

**Supplementary table 8: Table of correlation results clinical and neuroimaging**

| **Blink Characteristic** | **MDS-UPDRS III** | | | **MDS-UPDRS Facial Item 3.2** | | | | **MoCA** | | | | **Mean Putamen SBR** | | | **Mean Posterior Putamen SBR** | | | | **Putamen SBR Difference** | | | | | **Posterior Putamen SBR Difference** | | |
| --- | --- | --- | --- | --- | --- | --- | --- | --- | --- | --- | --- | --- | --- | --- | --- | --- | --- | --- | --- | --- | --- | --- | --- | --- | --- | --- |
|  | *r* | *p* | | *r* | | *p* | | | *r* | | *p* | *r* | *p* | | *r* | | *p* | | | *r* | | *p* | | *r* | *p* | |
| *Temporal Blink Metrics* | | | | | | | | | | | | | | | | | | | | | | | | | | |
| Blink Rate | **-0.37** | **<0.001** | | **-0,38** | | **<0.001** | | | 0,11 | | 0,23 | **0,32** | **<0.001** | | **0,35** | | **<0.001** | | | 0,12 | | 0,20 | | 0,06 | 0,54 | |
| Inter-Blink Interval | **0.25** | **<0.01** | | **0,29** | | **<0.01** | | | -0,08 | | 0,38 | **-0,30** | **<0.01** | | **-0,32** | | **<0.001** | | | -0,08 | | 0,39 | | -0,10 | 0,31 | |
| Interval Deviation | **0.31** | **<0.001** | | **0,29** | | **<0.01** | | | -0,06 | | 0,51 | **-0,23** | **<0.05** | | **-0,26** | | **<0.01** | | | -0,17 | | 0,08 | | -0,10 | 0,29 | |
| Relative Deviation | 0.18 | 0.06 | | 0,03 | | 0,72 | | | 0,00 | | 1,00 | 0,02 | 0,87 | | 0,00 | | 0,96 | | | -0,13 | | 0,17 | | -0,10 | 0,32 | |
| *Eyelid Kinematics Metrics* | | | | | | | | | | | | | | | | | | | | | | | | | | |
| Palpebral Aperture Shape | 0.17 | 0.06 | | 0,00 | | 0,96 | | | -0,01 | | 0,88 | -0,15 | 0,12 | | **-0,21** | | **<0.05** | | | 0,02 | | 0,87 | | 0,03 | 0,76 | |
| Palpebral Aperture Rigidity | **-0.35** | **<0.001** | | **-0,40** | | **<0.001** | | | 0,10 | | 0,29 | **0,30** | **<0.01** | | **0,28** | | **<0.01** | | | 0,04 | | 0,63 | | -0,03 | 0,71 | |
| Average Duration | 0.09 | 0.34 | | 0,13 | | 0,18 | | | 0,06 | | 0,52 | 0,07 | 0,48 | | 0,01 | | 0,90 | | | 0,08 | | 0,41 | | 0,09 | 0,33 | |
| Duration Variability | 0.03 | 0.77 | | -0,15 | | 0,13 | | | -0,07 | | 0,50 | 0,11 | 0,25 | | 0,13 | | 0,18 | | | 0,03 | | 0,80 | | 0,06 | 0,57 | |
| Average Amplitude | 0.03 | 0.74 | | **-0,19** | | **<0.05** | | | -0,02 | | 0,82 | -0,17 | 0,07 | | **-0,25** | | **<0.01** | | | -0,06 | | 0,55 | | -0,08 | 0,39 | |
| Amplitude Variability | -0.06 | 0.52 | | -0,17 | | 0,08 | | | -0,01 | | 0,92 | 0,06 | 0,56 | | 0,07 | | 0,48 | | | 0,07 | | 0,51 | | 0,08 | 0,40 | |
| *Eyelid Symmetry Assessment* | | | | | | | | | | | | | | | | | | | | | | | | | | |
| Average Duration Asymmetry | 0.12 | 0.20 | | **0,23** | | **<0.05** | | | -0,07 | | 0,49 | -0,07 | 0,47 | | -0,07 | | 0,48 | | | 0,03 | | 0,75 | | -0,01 | 0,95 | |
| Duration Asymmetry Variability | -0.02 | 0.84 | | -0,12 | | 0,22 | | | -0,10 | | 0,33 | 0,07 | 0,48 | | 0,04 | | 0,69 | | | 0,05 | | 0,61 | | 0,13 | 0,20 | |
| Average Amplitude Asymmetry | -0.02 | 0.85 | | -0,07 | | 0,44 | | | -0,06 | | 0,51 | 0,09 | 0,36 | | 0,02 | | 0,84 | | | 0,15 | | 0,12 | | **0,23** | **<0.05** | |
| Amplitude Asymmetry Variability | 0.17 | 0.09 | | 0,16 | | 0,10 | | | -0,05 | | 0,60 | -0,04 | 0,72 | | 0,07 | | 0,49 | | | 0,03 | | 0,77 | | -0,09 | 0,35 | |
|  |  | |  | |  | |  | | |  | |  | |  | |  | |  | | |  | |  | | |  |

**Supplemental References**

1. Kartynnik, Y., Ablavatski, A., Grishchenko, I. & Grundmann, M. Real-time Facial Surface Geometry from Monocular Video on Mobile GPUs. arXiv preprint, 10.48550/arXiv.1907.06724  (2019).
